# New Potent Membrane-Targeting Antibacterial Peptides from Viral Capsid Proteins

**DOI:** 10.3389/fmicb.2017.00775

**Published:** 2017-05-04

**Authors:** Susana A. Dias, João M. Freire, Clara Pérez-Peinado, Marco M. Domingues, Diana Gaspar, Nuno Vale, Paula Gomes, David Andreu, Sónia T. Henriques, Miguel A. R. B. Castanho, Ana S. Veiga

**Affiliations:** ^1^Instituto de Medicina Molecular, Faculdade de Medicina, Universidade de LisboaLisbon, Portugal; ^2^Department of Virology, Institut PasteurParis, France; ^3^Department of Experimental and Health Sciences, Pompeu Fabra University, Barcelona Biomedical Research ParkBarcelona, Spain; ^4^UCIBIO-REQUIMTE, Faculdade de Farmácia, Universidade do PortoPorto, Portugal; ^5^LAQV-REQUIMTE, Departamento de Química e Bioquímica, Faculdade de Ciências, Universidade do PortoPorto, Portugal; ^6^Institute for Molecular Bioscience, The University of Queensland, BrisbaneQLD, Australia

**Keywords:** antimicrobial peptides (AMPs), cell-penetrating peptides (CPPs), minimum inhibitory concentration (MIC), minimal bactericidal concentration (MBC), membrane permeabilization, atomic force microscopy (AFM)

## Abstract

The increasing prevalence of multidrug-resistant bacteria urges the development of new antibacterial agents. With a broad spectrum activity, antimicrobial peptides have been considered potential antibacterial drug leads. Using bioinformatic tools we have previously shown that viral structural proteins are a rich source for new bioactive peptide sequences, namely antimicrobial and cell-penetrating peptides. Here, we test the efficacy and mechanism of action of the most promising peptides among those previously identified against both Gram-positive and Gram-negative bacteria. Two cell-penetrating peptides, vCPP 0769 and vCPP 2319, have high antibacterial activity against *Staphylococcus aureus*, MRSA, *Escherichia coli*, and *Pseudomonas aeruginosa*, being thus multifunctional. The antibacterial mechanism of action of the two most active viral protein-derived peptides, vAMP 059 and vCPP 2319, was studied in detail. Both peptides act on both Gram-positive *S. aureus* and Gram-negative *P. aeruginosa*, with bacterial cell death occurring within minutes. Also, these peptides cause bacterial membrane permeabilization and damage of the bacterial envelope of *P. aeruginosa* cells. Overall, the results show that structural viral proteins are an abundant source for membrane-active peptides sequences with strong antibacterial properties.

## Introduction

Bacterial resistance to conventional antibiotics has increased drastically during the last decades, making difficult the effective treatment of infections caused by drug-resistant bacteria (Tanwar et al., 2014; Brown, 2015). According to a recent report from the World Health Organization (WHO, 2014), it is estimated that each year, in the European Union alone, over two million people become infected with resistant bacteria, of which 25,000 die. Also, it has been described that methicillin-resistant *Staphylococcus aureus* kills more Americans each year than HIV, Parkinson’s disease, emphysema, and homicide combined (Ventola, 2015). This represents a real threat to human health worldwide and has driven the search for novel effective antibacterial agents.

Antimicrobial peptides (AMPs) have emerged as potential alternatives to currently used antibiotics (Zasloff, 2002; Baltzer and Brown, 2011). This group of molecules can be found in all life forms, from microorganisms to plants and animals, and are known to have broad spectrum activity against multiple microorganisms, including bacteria, fungi, viruses and parasites (Zasloff, 2002; Jenssen et al., 2006; Nguyen et al., 2011). AMPs are very diverse in their amino acid sequences and folding (Hancock, 2001; Powers and Hancock, 2003; Peters et al., 2010), and display mechanisms of action that are distinct from the ones used by conventional antibiotics (Sancho-Vaello and Zeth, 2015). It has been proposed that the first step in their action involves the contact with bacterial membranes, which occurs through electrostatic and hydrophobic interactions with negatively charged lipids on the cell membrane (Yeaman and Yount, 2003; Teixeira et al., 2012). Membrane-targeting is usually followed by permeabilization and disruption of the lipid bilayer structure, leading to loss of integrity and ultimately cell death (Shai, 2002). Alternatively, some AMPs can exert their antimicrobial activity by targeting intracellular metabolic processes without damaging membrane integrity (Park et al., 2000; Brogden, 2005; Bahar and Ren, 2013). Currently, some AMPs are already clinically available, such as the cationic lipopeptide polymyxin B (Sandri et al., 2013) and the cyclic cationic peptide gramicidin S (Mogi and Kita, 2009), and many are in clinical development (Mahlapuu et al., 2016).

Peptide sequences with antimicrobial properties have been identified using different approaches, such as, identification of bioactive compounds on natural extracts (Bulet et al., 2004), *in silico* analysis of natural proteins (Torrent et al., 2012), *de novo* or structure-based design, or chimeras of peptide fragments (Piers et al., 1994; Wu and Hancock, 1999; Rozek et al., 2003; Brogden and Brogden, 2011). Since enveloped viruses are abundant in multifunctional proteins, using bioinformatic tools we searched structural viral proteins for bioactive peptides sequences (Freire et al., 2015a), namely AMPs and cell-penetrating peptides (CPPs). CPPs are a group of peptides capable of crossing biological membranes without causing significant membrane damage (Gräslund et al., 2011). Given the similarities in the structure and activity between these two groups of peptides, it has been proposed that CPPs are not totally distinct from AMPs (Henriques et al., 2006; Zhu et al., 2006; Splith and Neundorf, 2011).

In this study, we intended to investigate the antibacterial activity and mechanism of action of previously identified viral protein-derived peptides (**Table 1**) (Freire et al., 2015a). First, the potential antibacterial activity of six selected viral protein-derived peptides proven to have cell-penetrating properties (vCPPs) was evaluated against Gram-positive and Gram-negative bacteria. The results obtained allowed to identify two vCPPs with high antibacterial activity, vCPP 0769 and vCPP 2319, showing that viral protein-derived sequences are a possible source for peptides with dual action. Furthermore, the antibacterial mechanism of action of the two most active viral protein-derived peptide sequences, vAMP 059, previously identified (Freire et al., 2015a), and vCPP 2319, was investigated and the results show that the peptides act mainly through a mechanism involving membrane disruption. This study shows the potential of viral structural proteins as a source of bioactive peptides sequences with antibacterial properties.

**Table 1:** Viral protein-derived peptides used in this study.

Name	Sequence	Length	Net charge	Source (protein:position)
vCPP 0275	KKRYKKKYKAYKPYKKKKKF-NH_2_	20	+14	Cauliflower mosaic virus (Capsid: aa367–387)
vCPP 0417	SPRRRTPSPRRRRSQSPRRR-NH_2_	20	+11	Hepatitis B virus genotype C (Capsid: aa155–175)
vCPP 0667	RPRRRATTRRRITTGTRRRR-NH_2_	20	+12	Human Adenovirus C serotype 1 (Minor Core Protein – Capsid: aa314–334)
vCPP 0769	RRLTLRQLLGLGSRRRRRSR-NH_2_	20	+10	Fowl adenovirus A serotype 1 (Major Capsid Protein: aa17–37)
vCPP 1779	GRRGPRRANQNGTRRRRRRT-NH_2_	20	+11	Barley Virus (Capsid: aa5–25)
vCPP 2319	WRRRYRRWRRRRRWRRRPRR-NH_2_	20	+16	Torque teno douroucouli vírus (Capsid: aa16–36)
vAMP 059	INWKKWWQVFYTVV-NH_2_	14	+3	Rotavirus VP7 (Capsid: aa94–107)


## Materials and Methods

### Peptide Selection and Synthesis

Peptide selection was performed based on a previous study (Freire et al., 2015a) in which viral proteins were searched for AMPs and CPPs using AMPA and CellPPD bioinformatic tools, respectively. The AMPA server (Torrent et al., 2012) is based on an antimicrobial propensity scale that considers the physical chemical properties of each amino acid, such as hydrophobicity and amphipathicity, and the relevance of amino acid position for antimicrobial activity. An antimicrobial index (AI) < 0.225 was considered a positive hit for an AMP. CellPPD is a Support Vector Machine (SVM) that scores each amino acid residue sequence with a SVM score (Gautam et al., 2013). A SVM > 0 was considered a positive CPP hit. Several peptide sequences were selected for experimental validation, based on their sequence novelty (when compared to the AMP/CPP sequences listed on the existing databases) and best ranking in AI and SVM score. The selection covered a broad range of scores and peptide sequence physical–chemical properties such as hydrophobicity and amphipathicity. The most active peptides were selected for the present study.

All the peptides have an amidated C-terminus and a free amine N-terminus. The viral protein-derived CPPs (vCPPs) used in this study, **Table 1**, were synthesized by Bachem AG (Bubendorf, Switzerland) with a purity of >95%. The viral protein-derived AMP used, vAMP 059 (**Table 1**), was synthesized on a standard Fmoc-Rink amide MBHA resin, using a Liberty 1 Microwave Peptide Synthesizer (CEM Corporation, Mathews, NC, USA). Standard Fmoc/tBu SPPS protocols were applied following procedures previously reported by us (Monteiro et al., 2015; Barbosa et al., 2017). The crude peptide was purified by preparative HPLC to a final 100% purity, as evaluated by analytical HPLC. The pure peptide was analyzed by electrospray-ionization/ion-trap mass spectrometry (ESI-IT MS). The average calculated mass and observed mass values of all vCPPs and vAMP 059 are shown in Supplementary Table S1.

To prepare stock solutions lyophilized peptides were weighed out on a high precision analytical microbalance (0.01 mg ± 0.02), dissolved in sterile Milli-Q water and stored at –20°C.

### Antibacterial Activity Assay

The antibacterial activity of the vCPPs was tested using a standard broth microdilution procedure (Wikler et al., 2006; Wiegand et al., 2008) to evaluate the bacterial growth inhibition and determine the minimal inhibitory concentration (MIC). The assay was performed using the following strains: Gram-positive *Staphylococcus aureus* (*S. aureus*) ATCC 25923 and methicillin-resistant *Staphylococcus aureus* (MRSA) ATCC 33591, and Gram-negative *Escherichia coli* (*E. coli*) ATCC 25922 and *Pseudomonas aeruginosa* (*P. aeruginosa*) ATCC 27853, obtained from American Type Culture Collection (ATCC) (Manassas, VA, USA). Bacterial suspensions were prepared by direct suspension of morphologically similar colonies on Mueller Hinton Broth (MHB) from BD (Franklin Lakes, NJ, USA) to a final concentration of 1 × 10^6^ CFU/mL, and added to a sterile 96-well microtiter polypropylene plate from Corning (Corning, NY, USA) containing two-fold dilutions of each peptide. Final bacterial concentration was 5 × 10^5^ CFU/mL whereas peptide concentrations ranged between 100 and 0.78 μM. The 96-well plate was incubated at 37°C for 18 h. The MIC was defined as the lowest peptide concentration required to inhibited visible bacterial growth. The assay was performed in triplicate.

### Bactericidal Activity Assay

To study the bactericidal activity of the most active viral protein-derived peptides, the minimal bactericidal concentration (MBC) was determined by a colony count assay (Barry et al., 1999). After the MIC assay, aliquots were removed from the wells with no visible bacterial growth, serially diluted in MHB, plated on nutrient-rich trypcase soy agar (TSA) plates from bioMérieux (Marcy l’Etoile, France), and incubated at 37°C for 24 h. After incubation, bacterial colonies were counted and the MBC was defined as the lowest peptide concentration that caused ≥99.9% cell death of the initial bacterial inoculum. The assay was performed in triplicate.

### Time-Kill Assay

For viral protein-derived peptides displaying bactericidal activity, bacterial killing kinetics was determined by a conventional method based on colony counts obtained after different times of exposure of the bacterial cells to the peptides (Barry et al., 1999). The assay was performed using *S. aureus* and *P. aeruginosa* as representative strains of Gram-positive and Gram-negative bacteria, respectively. Bacterial suspensions prepared in MHB at 5 × 10^5^ CFU/mL were treated with each peptide at final concentrations corresponding to their MBC, and incubated at 37°C and 200 rpm. A control of bacterial suspensions without peptide was performed under the same conditions. Aliquots of untreated and peptide-treated bacterial suspensions were withdrawn at 0, 15, 30, 60, 120, and 180 min, serially diluted in MHB, and plated on TSA plates. Bacterial colonies were counted after 24 h of incubation at 37°C. Viable bacteria (in CFU/mL) are reported as percentage of the control. The assay was performed in triplicate.

### SYTOX Green Uptake Assay

SYTOX^®^ Green (Invitrogen, Carlsbad, CA, USA) is a high-affinity nucleic acid stain that only penetrates cells with compromised plasma membranes and emits fluorescence when bound to DNA (Roth et al., 1997). A SYTOX Green uptake assay (Torcato et al., 2013; Sani et al., 2015) was performed using *P. aeruginosa* to study the effect of viral protein-derived peptides on those bacterial membranes. Bacterial suspensions at 1 × 10^8^ CFU/mL in MHB were centrifuged for 10 min at 4000 × *g* and the pellet resuspended in 10 mM HEPES buffer, pH 7.4, containing 150 mM NaCl, to a final concentration of 5 × 10^5^ CFU/mL. The bacterial suspensions were incubated for 1 h at 37°C and 200 rpm, with twofold dilutions of each bactericidal viral protein-derived peptide in a range of concentrations starting at their corresponding MBC. Peptide-treated bacterial cells were incubated with 0.1 μM of SYTOX Green for 10 min, on ice and protected from light. The fluorescence emission intensity signal was measured by flow cytometry (excitation with 488 nm laser and detection at 530 nm with 30 nm bandpass) and a total of 50 000 events were recorded. Flow cytometry experiments were performed using a BD LSR Fortessa from BD Biosciences (San Jose, CA, USA). The percentage of permeabilized cells was determined considering that untreated bacterial cells (negative control) are 0% permeabilized and bacterial cells treated with isopropyl alcohol (positive control) are 100% permeabilized. To study the correlation between bacterial cell permeabilization and viability, a viability assay using a colony count method was used. Using the same conditions described for the SYTOX Green uptake assay, untreated and peptide-treated bacterial suspensions were incubated, aliquots were serially diluted in HEPES buffer, and plated on TSA plates. Plates were incubated at 37°C for 24 h. From the number of bacterial colonies obtained, viable bacteria (in CFU/mL) are reported as percentage of the control. The assays were performed in triplicate.

### Atomic Force Microscopy (AFM) Imaging

Atomic force microscopy (AFM) imaging was used to study the effect of the bactericidal viral protein-derived peptides on *P. aeruginosa* cells. Bacterial suspensions at 1 × 10^8^ CFU/mL in MHB were centrifuged for 10 min at 4000 × *g*, and the pellet resuspended in HEPES buffer to a final concentration of 1 × 10^7^ CFU/mL. The bacterial suspensions were treated with each peptide at concentrations corresponding to their MBC, as well as the concentrations above and below their MBC, and incubated for 1 h at 37°C and 200 rpm. As control, untreated bacterial suspension was also incubated using the same conditions. A 200 μL droplet of each test sample was applied on a poly-L-lysine-coated glass slide and allowed to deposit for 20 min at room temperature. Each sample was then rinsed 10 times with sterile Milli-Q water and allowed to dry for 30 min at room temperature. AFM images were acquired using a JPK NanoWizard II (Berlin, Germany) mounted on a Zeiss Axiovert 200 inverted microscope (Göttingen, Germany). Measurements were carried out in air and in intermittent contact mode using uncoated silicon ACL cantilevers from APPNano (Mountain View, CA, USA). ACL cantilevers had typical resonance frequencies of 190 kHz and a spring constant of 45 N/m. Bacteria were first visualized through the optical microscope before being selected for imaging. On average, 14 untreated bacteria and 10 individual bacterial cells were imaged for each peptide concentration, with a total area of 4 μm × 4 μm from three independent bacterial preparation slides. Height images were recorded and treated with JPK SPM data processing 5.1.8.

## Results

### Antibacterial Activity of vCPPs

The antibacterial activity of six viral protein-derived CPPs (vCPPs; **Table 1**) was evaluated by determining the MIC against *S. aureus* and MRSA, and *E. coli* and *P. aeruginosa*, as representative models of Gram-positive and Gram-negative bacteria, respectively. The MIC values obtained for each peptide are presented in **Table 2**. Among the vCPPs studied, two peptides, vCPP 0769 and vCPP 2319, exhibited high antibacterial activity against all bacterial strains tested. vCPP 0769 was particularly active against *S. aureus*, MRSA and *P. aeruginosa*, with a MIC of 3.13 μM for these three bacteria, whereas for *E. coli* a MIC value of 25 μM was obtained. vCPP 2319 displayed high antibacterial activity against all bacterial strains tested, with a MIC of 1.56 μM for *S. aureus* and MRSA, and 3.13 μM for *E. coli* and *P. aeruginosa*.

**Table 2:** Antibacterial activity of vCPPs.

Peptide	MIC (μM)			
	
	*S. aureus*	MRSA	*E. coli*	*P. aeruginosa*
vCPP 0275	25–50	50	12.5	100
vCPP 0417	>100	>100	25	100
vCPP 0667	50	100	12.5	25
vCPP 0769	3.13	3.13	25	3.13
vCPP 1779	100–>100	>100	25	25
vCPP 2319	1.56	1.56	3.13	3.13


### Bactericidal Activity of Viral Protein-Derived Peptides

To assess whether vCPP 0769 and vCPP 2319 are bactericidal or bacteriostatic, their MBC was determined for all bacterial strains tested. Peptides are considered bacteriostatic if their MBC is more than fourfold their MIC, and bactericidal when their MBC is equal or lower than four-fold their MIC (Levison and Levison, 2009). The MBC values obtained are presented in **Table 3**. vCPP 2319 exhibited bactericidal activity against all bacterial strains tested. On the other hand, vCPP 0769 showed to be bactericidal only against *E. coli* and *P. aeruginosa*, whereas for *S. aureus* and MRSA it only inhibited bacterial growth. Additionally, the bactericidal activity of the peptide vAMP 059 was also studied. This peptide was identified in our previous screen for antimicrobial sequences from structural viral proteins showing high antibacterial activity against *S. aureus* and *P. aeruginosa*, with MIC values of 0.78 and 6.25 μM, respectively (Freire et al., 2015a). MBC values showed the peptide is bactericidal against both *S. aureus* and *P. aeruginosa* strains tested.

**Table 3:** Bactericidal activity of vCPP 0769, vCPP 2319, and vAMP 059.

Peptide	MBC (μM)			
	
	*S. aureus*	MRSA	*E. coli*	*P. aeruginosa*
vCPP 0769	>100	>100	50	6.25
vCPP 2319	3.13	3.13	3.13	3.13
vAMP 059	1.56	–	–	6.25


### Bactericidal Kinetics of Viral Protein-Derived Peptides

**FIGURE 1:**
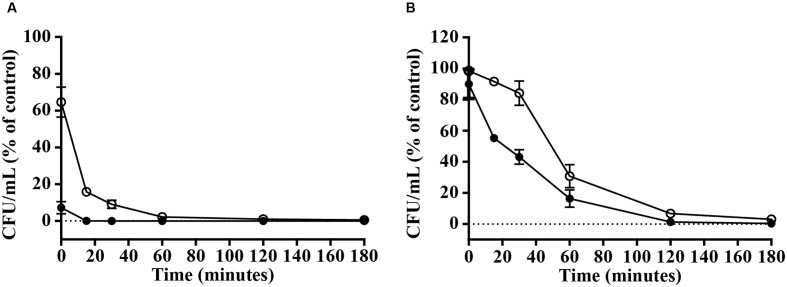
**Killing kinetics of *S. aureus*** **(A)** and *P. aeruginosa* **(B)**. Bacterial cells were exposed to the peptides vAMP 059 (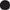) and vCPP 2319 (∘) at their MBC. Viable bacteria (in CFU/mL) are reported as percentage of the control without peptide. Data correspond to mean ± SD of three independent experiments.

The above-mentioned results show that vAMP 059 and vCPP 2319 are the most active peptides with a broad bactericidal activity against the Gram-positive and Gram-negative bacterial strains tested. Bacterial killing kinetics of *S. aureus* and *P. aeruginosa* by vAMP 059 and vCPP 2319 were determined at the MBC (**Figure 1**). In the presence of both peptides, a significant reduction in the number of viable bacteria occurred within minutes, although a faster bacterial killing was observed for *S. aureus* (**Figure 1A**) when compared to *P. aeruginosa* (**Figure 1B**). Both vAMP 059 and vCPP 2319 exhibited a fast bactericidal activity against *S. aureus* with a reduction in bacterial viability of 99.9 and 90.9%, respectively, after 30 min of peptide activity. Both peptides exhibited a slower bactericidal activity against *P. aeruginosa*. In this case, cell viability decreased more gradually over time with a 99.7 and 97% reduction of bacterial viability occurring after 180 min of exposure to vAMP 059 and vCPP 2319, respectively.

### Viral Protein-Derived Peptides Induce Permeabilization of *P. aeruginosa* Cells

**FIGURE 2:**
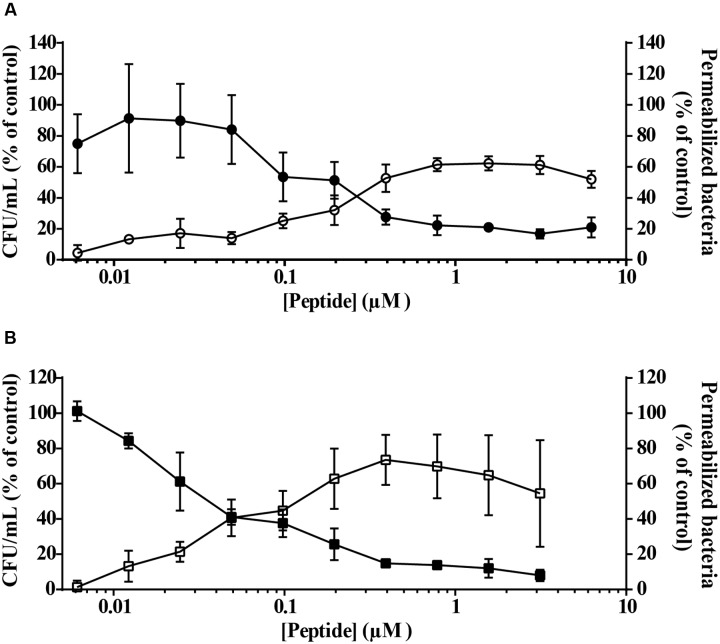
**Bacterial cells viability and membrane permeabilization after treatment with vAMP 059** **(A)** or vCPP 2319 **(B)**. *P. aeruginosa* cells were incubated with a range of concentrations of vAMP 059 or vCPP 2319 for 1 h at 37°C. Viable bacteria (in CFU/mL) (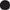, 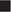) and permeabilized bacteria (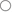, 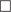) are reported as a percentage of the control. Data correspond to mean ± SD of three independent experiments.

A fast bacterial killing kinetics is frequently observed for AMPs and is related to their membrane-disrupting mode of action. To investigate if peptides vAMP 059 and vCPP 2319 affect bacterial membrane integrity, *P. aeruginosa* permeability to the SYTOX Green dye was studied after treatment with increasing peptide concentrations, with an increase in the dye fluorescence intensity directly reflecting membrane permeabilization (Roth et al., 1997). Results (**Figure 2**) showed an increase in membrane permeabilization with increasing peptide concentrations. However, it was also noticed that at high concentrations of both peptides there was a slight reduction in permeabilization. Such result may be due to SYTOX Green displacement from nucleic acids by the peptides, which would cause a reduction in its fluorescence emission intensity and consequently an apparent reduction in the percentage of permeabilized bacteria.

Simultaneously with the SYTOX Green uptake assay, a viability assay was performed for both peptides under the same conditions. Results demonstrated a correlation between *P. aeruginosa* permeability and viability in the presence of vAMP 059 (**Figure 2A**) and vCPP 2319 (**Figure 2B**). These results show that both peptides kill bacteria through membrane-disruption mechanisms.

### Bioimaging of the Viral Protein-Derived Peptides Effect on *P. aeruginosa* Cells

**FIGURE 3:**
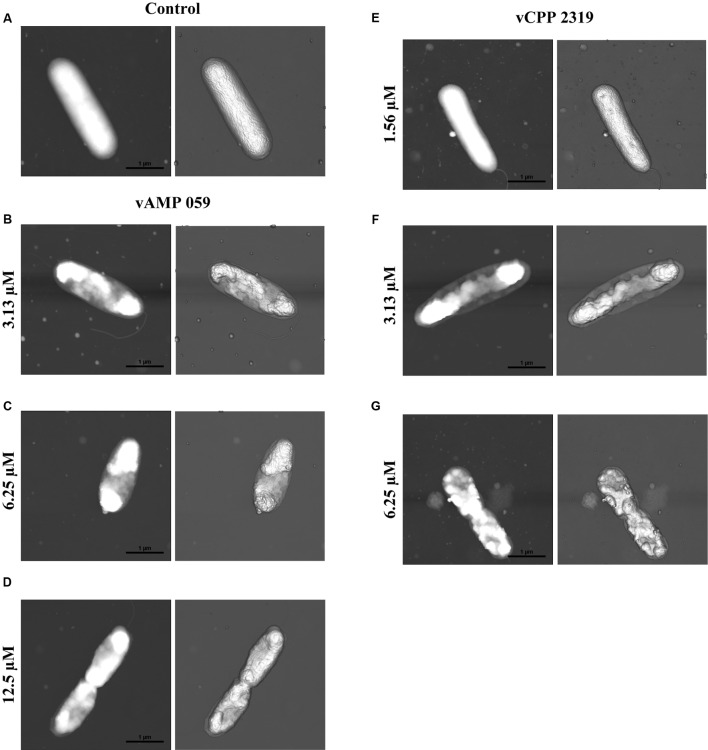
**Effect of vAMP 059 and vCPP 2319 on *P. aeruginosa* cells imaged by AFM.** **(A)** AFM height image (left image) and three-dimensional projection (right image) of *P. aeruginosa* cells incubated for 1 h in the absence of peptide (control). **(B–G)** AFM height images and three-dimensional projections of *P. aeruginosa* cells incubated for 1 h with increasing concentrations of vAMP 059 **(B–D)** and vCPP 2319 **(E–G)**. Total scanning area for each image was 4 μm × 4 μm.

Atomic force microscopy imaging was used to visualize the morphological changes in *P. aeruginosa* after treatment with vAMP 059 and vCPP 2319. The images obtained in air for untreated *P. aeruginosa* cells (**Figure 3A**) showed, in all cases, the characteristic rod shaped structure as well as a corrugated surface with no visible pores or ruptures. Treatment of bacteria with increasing concentrations of both peptides revealed a clear effect on cell envelope integrity when compared with untreated bacteria, although the cells maintained their global rod-like form. After treatment with vAMP 059, a pronounced collapse in *P. aeruginosa* cell envelope occurred at all concentrations tested (**Figures 3B–D**). For vCPP 2319 a similar result was observed, but a more gradual effect with increasing concentrations of peptide was observed (**Figures 3E–G**). At 1.56 μM, a concentration below MBC, only minor alterations on bacterial topography were observed.

## Discussion

The therapeutic options for treatment of bacterial infections are drastically decreasing due to a fast increase in drug resistance. Driven by the need to discover new and effective antimicrobial agents, this work has focused on a group of promising alternatives, the AMPs.

In a previous study performed in our lab, we became aware that, among AMPs and CPPs derived from natural sources and listed on existing databases, only a few of them were related to viral proteins. Using bioinformatic tools, a set of peptide sequences with potential antimicrobial and cell-penetrating properties was identified, suggesting that viruses are an underexplored source for AMPs and CPPs (Freire et al., 2015a). One viral protein-derived AMP (vAMP 059) and ten viral protein-derived CPPs (vCPPs) were identified. In this study, from the six more active vCPPs, we were able to identify two peptides, vCPP 0769 and vCPP 2319, with concurrent, strong antibacterial activity. vCPP 2319 demonstrated to be active at low micromolar range concentrations, being bactericidal against all the bacterial strains tested. vCPP 0769 revealed high antibacterial activity against the *S. aureus*, MRSA and *P. aeruginosa* strains, and a moderate activity against *E. coli*, but only displayed bactericidal activity against the Gram-negative bacterial strains tested. Therefore, both peptides reveal a dual cell-penetrating and antibacterial activity, a peculiar cross functionality that has been reported for few other peptides (Takeshima et al., 2003; Nekhotiaeva et al., 2004; Bobone et al., 2011). It has been proposed that peptides with dual antimicrobial and cell-penetrating properties might constitute potential candidates to target intracellular pathogenic bacteria (Bahnsen et al., 2013; Torcato et al., 2013) that escape host defenses (Ernst et al., 1999) and are extremely difficult to treat with conventional antibiotics (Clement et al., 2005).

In the present study, the results also show that the previously identified viral protein-derived peptide vAMP 059, with demonstrated activity against *S. aureus* and *P. aeruginosa* (Freire et al., 2015a), revealed a bactericidal mode of action against these bacterial strains. It is interesting to notice that this peptide, as well as both vCPP 0769 and vCPP 2319, are derived from viral capsid proteins (Freire et al., 2015a). Despite the fact that only few viral-derived AMPs are known, there are other examples of peptides with antimicrobial properties derived from viral capsid proteins. HBc ARD, is a peptide derived from the capsid protein of the human hepatitis B virus (Chen et al., 2013), and pepR, derived from the putative RNA-binding domain of the dengue virus capsid protein (Alves et al., 2010). Viral capsid proteins are known as multifunctional proteins and some of them can also be described as super charged proteins, a class of proteins with high net charge to molecular mass ratio and are known to have cell-membrane translocating properties (Freire et al., 2015b). In fact, there are several examples of cell-penetrating domains found in capsid proteins. With Dengue virus capsid protein as an example, we showed that one of these domains, the aforementioned pepR, also displays antibacterial activity. This potential antibacterial activity of viral proteins may have conferred viruses an evolutionary advantage over bacteria (Freire et al., 2015b). Additionally, the presence of domains with both antibacterial and cell-penetrating properties in viral capsid proteins demonstrates the potential use of viral proteins in drug discovery.

To get insights on the mode of action of the viral protein-derived peptides, the most active peptides vAMP 059 and vCPP 2319 were studied. Both peptides revealed a fast killing kinetics against *S. aureus* and *P. aeruginosa*. Since it is known that antimicrobial agents that kill within minutes of bacterial exposure, at concentrations similar to their MICs, act by disrupting bacterial membranes (Zasloff, 2002; Melo et al., 2009), we can suggest that both viral protein-derived peptides induce bacterial death through membrane disruption. This behavior is common amongst AMPs; for instance, the antimicrobial peptide melittin, derived from bee venom, exhibits an extremely rapid membrane-disrupting action, killing bacteria within minutes (Kaplan et al., 2011). Additionally, when comparing vAMP 059 and vCPP 2319 killing kinetics on both bacterial strains tested here, faster rates were observed against *S. aureus* than against *P. aeruginosa.* This behavior can be related to the differential peptide affinity for the molecular structures on Gram-negative or Gram-positive bacteria envelope surfaces. Gram-positive bacteria envelope consists on a single lipid membrane followed by a thick peptidoglycan layer enriched in negatively charged teichoic acids (Xia et al., 2010). In contrast, Gram-negative bacteria possesses an inner cytoplasmic membrane surrounded by a thin peptidoglycan layer and an outer membrane containing negative lipopolysaccharides (LPS) (Beveridge, 1999). For instance, the peptide rBPI_21_ exerts its antimicrobial activity through interactions with LPS of the outer membrane of Gram-negative bacteria, followed by fusion of the bacterial outer and inner membranes (Domingues et al., 2009). In turn, the antimicrobial peptide omiganan has high affinity for the peptidoglycan layer in Gram-positive bacteria (Melo and Castanho, 2007). vAMP 059 and vCPP 2319 may have faster interaction with peptidoglycan when compared to LPS, resulting on a faster killing kinetics towards *S. aureus* rather than *P. aeruginosa*. The hypothesis that vAMP 059 and vCPP 2319 cause bacterial cell membrane disruption was confirmed in Gram-negative *P. aeruginosa* cells. Both peptides induce permeabilization of the outer and inner membranes, in a concentration-dependent manner, as shown using the non-permeable SYTOX Green dye, which was able to bind to the nucleic acids of bacterial cells treated with peptide. A mechanism involving membrane disruption was further confirmed by AFM imaging. Treatment of *P. aeruginosa* cells with vAMP 059 induced a pronounced collapse at the septal region of the cell envelope at all concentrations tested. Negatively charged cardiolipin domains are located at the apical and septal regions of *E. coli* cells (Mileykovskaya and Dowhan, 2000). Assuming a similar cardiolipin-distribution for *P. aeruginosa*, the observed collapse in the mid-region could be explained by accumulation of the peptides in these regions due to electrostatic attractions between the positively charged peptides and these negatively charged domains. Treatment of *P. aeruginosa* with vCPP 2319 at concentrations below the MBC did not cause substantial alterations on cell membrane morphology. However, treatment with MBC, or higher, caused a marked collapse of the membrane structure. Overall, AFM imaging suggested that both peptides cause significant structural alterations on bacterial cell surface, which is in agreement with the proposed hypothesis that vAMP 059 and vCPP 2319 act at the membrane-level.

## Author Contributions

SD, CP-P, MD, DG, ST-H, MC, and ASV designed the experiments. SD, CP-P, MD, DG, and ASV performed the experimental work and data analysis. JF contributed with the design of the studied peptides. PG and NV contributed with peptide synthesis. All authors contributed to data interpretation and discussion. SD, MC, and ASV wrote the manuscript with contributions from all other authors.

## Conflict of Interest Statement

The authors declare that the research was conducted in the absence of any commercial or financial relationships that could be construed as a potential conflict of interest.
