# Supplementary material for: New Potent Membrane-Targeting Antibacterial Peptides from Viral Capsid Proteins
Source: Front Microbiol. 2017 May 4;8:775. doi: 10.3389/fmicb.2017.00775 (PMC5415599; doi:10.3389/fmicb.2017.00775)
Supplement: Supplementary file 1 [file Table_1.docx]

Supplementary Material

**New potent membrane-targeting antibacterial peptides from viral capsid proteins**

**Susana Almeida Dias^1^, João Miguel Freire^1,2^, Clara Pérez-Peinado^3^, Marco M. Domingues^1^, Diana Gaspar^1^, Nuno Vale^4^, Paula Gomes^5^, David Andreu^3^, Sónia Troeira Henriques^6^, Miguel A. R. B. Castanho^1^, Ana Salomé Veiga^1*^**

*** Correspondence:**Ana Salomé Veiga
aveiga@medicina.ulisboa.pt

# Supplementary Table

**Table 1: Average calculated mass and observed mass of the viral protein-derived peptides used in this study.**

| **Peptide** | **Average Calculated Mass**  **(Da)**^a^ | **Observed Mass (Da)** |
| --- | --- | --- |
| **vCPP 0275** | 2679.38 | 2679.86^b^ |
| **vCPP 0417** | 2544.92 | 2545.43^b^ |
| **vCPP 0667** | 2579.03 | 2579.38^b^ |
| **vCPP 0769** | 2506.01 | 2506.12^b^ |
| **vCPP 1779** | 2476.81 | 2477.59^b^ |
| **vCPP 2319** | 3178.78 | 3178.27^b^ |
| **vAMP 059** | 1896.30 | 1895.72^c^ |

^a^: average calculated mass from the amino acid sequence

^b^: observed mass as calculated from Matrix-Assisted Laser Desorption/Ionization Mass Spectrometry (MALDI-MS)

^c^: observed mass as calculated from ElectroSpray-Ionization/Ion-Trap Mass Spectrometry (ESI-IT MS)
